# Supplementary material for: New SARS-CoV-2 Infection Detected in an Italian Pet Cat by RT-qPCR from Deep Pharyngeal Swab
Source: Pathogens. 2020 Sep 11;9(9):746. doi: 10.3390/pathogens9090746 (PMC7559392; doi:10.3390/pathogens9090746)
Supplement: Supplementary file 1 [file pathogens-09-00746-s001.zip › Figure S1.pdf]

Figure S1. Amplification profiles for the detection of SARS-CoV-2.

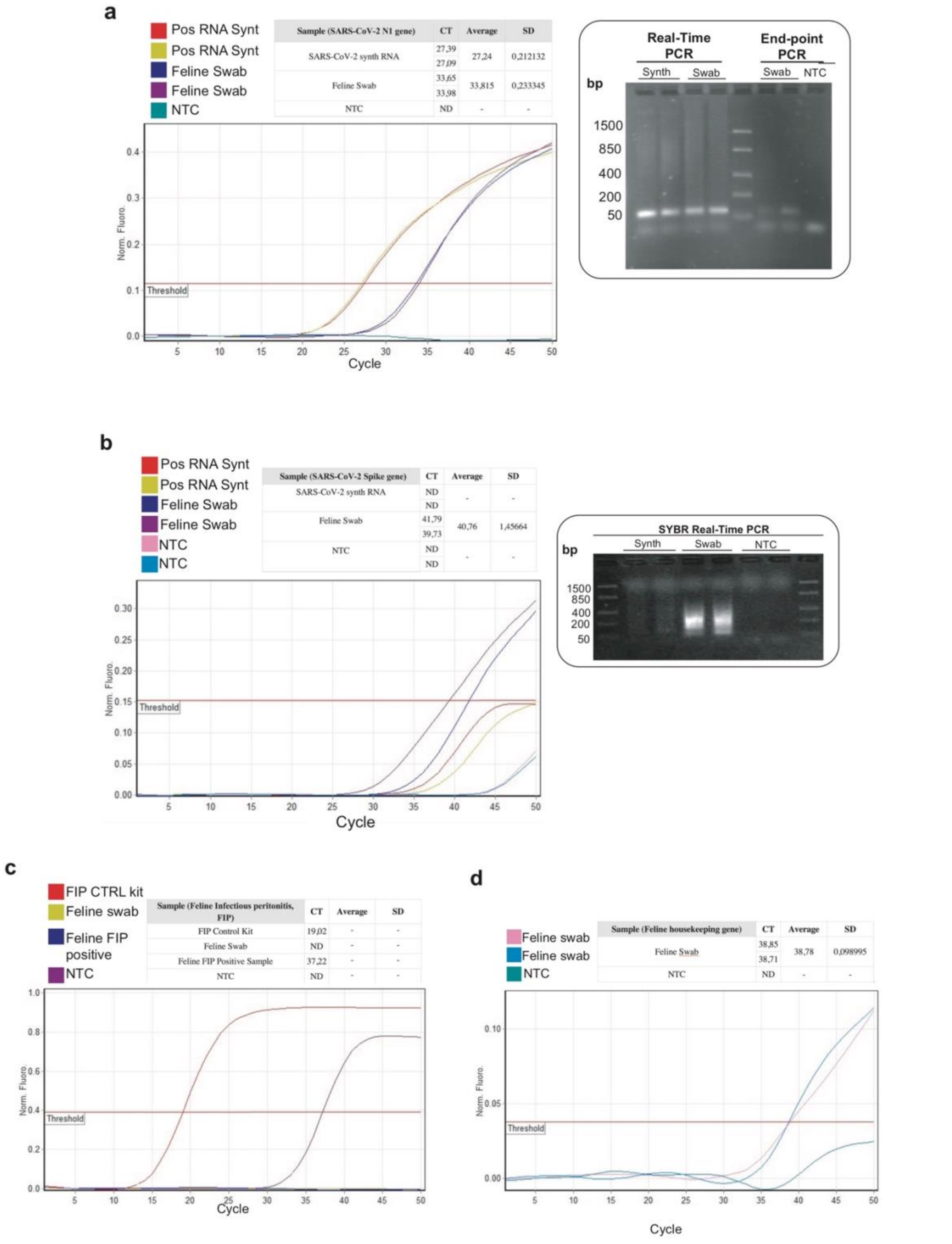

Sample (SARS-CoV-2 N1 gene)	CT	Average	SD
SARS-CoV-2 synth RNA	27,39<br>27,09	27,24	0,212132
Feline Swab	33,65<br>33,98	33,815	0,233345
NTC	ND	-	-

Sample (SARS-CoV-2 Spike gene)	CT	Average	SD
SARS-CoV-2 synth RNA	ND<br>ND	-	-
Feline Swab	41,79<br>39,73	40,76	1,45664
NTC	ND<br>ND	-	-

Sample (Feline Infectious peritonitis, FIP)	CT	Average	SD
FIP Control Kit	19,02	-	-
Feline Swab	ND	-	-
Feline FIP Positive Sample	37,22	-	-
NTC	ND	-	-

Sample (Feline housekeeping gene)	CT	Average	SD
Feline Swab	38,85<br>38,71	38,78	0,098995
NTC	ND	-	-

CT: cycle threshold, **SD:** standard deviation, **ND:** not detected, **NTC:** no template control.

a) RT-qPCR detection of SARS-CoV-2 N1 gene (left) and relative real-time and End-point PCR in 1,8% agarose gel (right); **b)** RT-qPCR detection of SARS-CoV-2 spike gene (left) and respective agarose fragment evaluation SYBR PCR (right). **c)** RT-qPCR detection of FIP; **d)** RT-qPCR detection of feline housekeeping gene (Techne-FCoV Kit).
