# Supplementary figures and images for: New SARS-CoV-2 Infection Detected in an Italian Pet Cat by RT-qPCR from Deep Pharyngeal Swab
Source: Pathogens. 2020 Sep 11;9(9):746. doi: 10.3390/pathogens9090746 (PMC7559392; doi:10.3390/pathogens9090746)

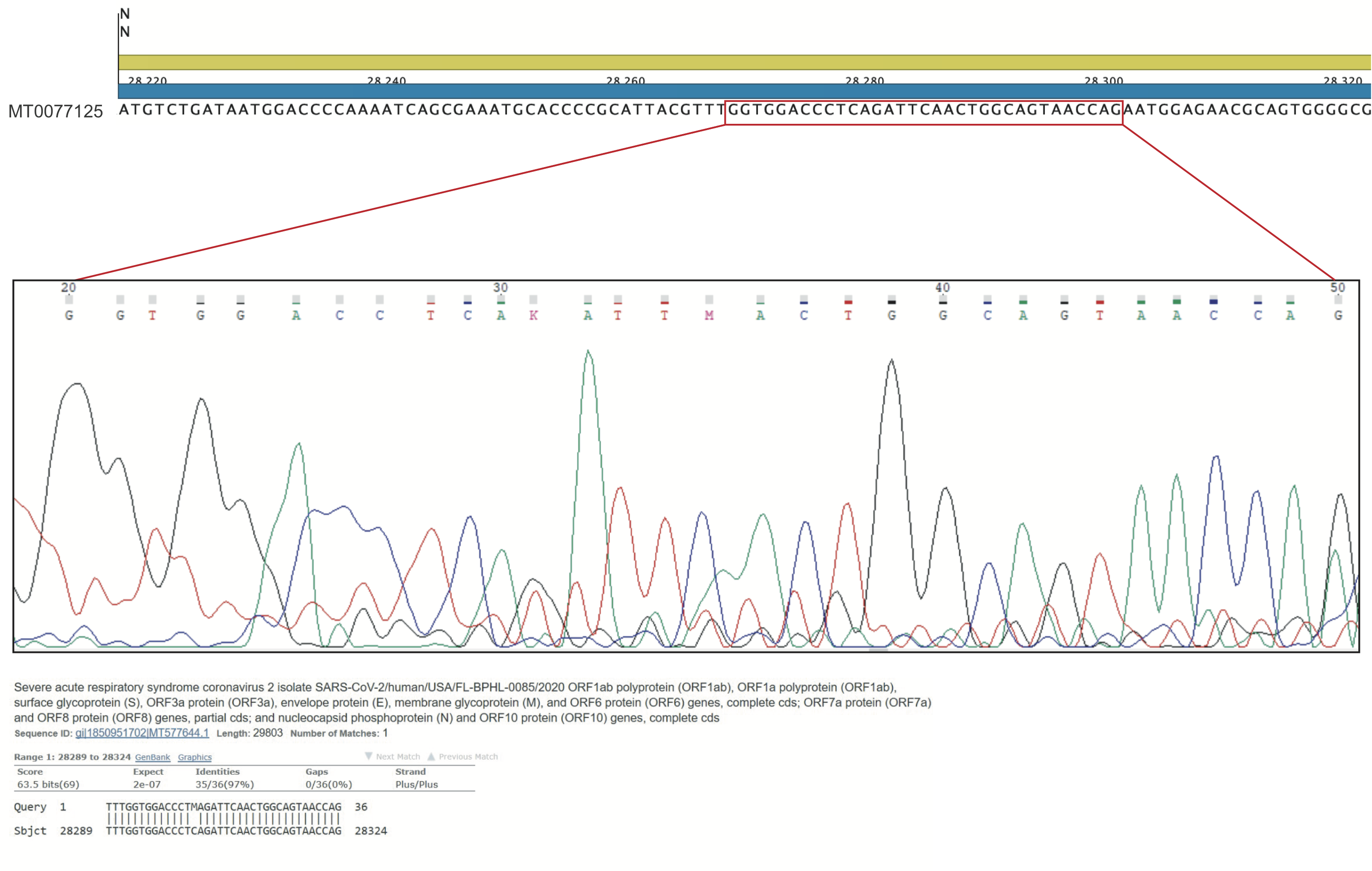

Supplement: Supplementary file 1 [file pathogens-09-00746-s001.zip › FigS2.tiff]
